# Revisiting Telomere Shortening in Cancer

**DOI:** 10.3390/cells8020107

**Published:** 2019-01-31

**Authors:** Keiji Okamoto, Hiroyuki Seimiya

**Affiliations:** Division of Molecular Biotherapy, Cancer Chemotherapy Center, Japanese Foundation for Cancer Research, Koto-ku, Tokyo 135-8550, Japan; keiji.okamoto@jfcr.or.jp

**Keywords:** telomere, telomerase, TERT, cancer, interferon stimulated gene, telomere position effect, shelterin, TERRA, biomarker

## Abstract

Telomeres, the protective structures of chromosome ends are gradually shortened by each cell division, eventually leading to senescence or apoptosis. Cancer cells maintain the telomere length for unlimited growth by telomerase reactivation or a recombination-based mechanism. Recent genome-wide analyses have unveiled genetic and epigenetic alterations of the telomere maintenance machinery in cancer. While telomerase inhibition reveals that longer telomeres are more advantageous for cell survival, cancer cells often have paradoxically shorter telomeres compared with those found in the normal tissues. In this review, we summarize the latest knowledge about telomere length alterations in cancer and revisit its rationality. Finally, we discuss the potential utility of telomere length as a prognostic biomarker.

## 1. Introduction

Telomeres are protective structures of chromosome ends containing repetitive DNA sequences (TTAGGG in vertebrates) and associated proteins. In human somatic cells, telomeres are gradually shortened by each cell division by semi-conservative DNA replication, and cells with extremely shortened telomeres reach senescence or apoptosis. However, self-renewing cells, such as embryonic stem cells and most cancer cells, can maintain telomeres to overcome the cell senescence or apoptosis caused by telomere shortening and acquire immortality. Two mechanisms for telomere maintenance have been discovered in previous studies: Transcriptional activation of a telomere reverse transcriptase called telomerase and the activation of alternative lengthening of telomeres (ALT), which is a telomerase-independent telomere maintenance mechanism utilizing the DNA homologous recombination repair pathway. Of all cancer cells, 85–95% expresses telomerase, whereas ~5–15% exhibit ALT pathway activation [1,2].

Telomerase consists of two essential components, telomerase RNA (TERC/TR) and telomere reverse transcriptase (TERT), and some accessory proteins, such as DKC1, NHP2, NOP10, pontin/reptin and TCAB1 [3]. Telomerase is recruited to single stranded telomeric DNA through interaction with the telomere-localizing protein TPP1 [4,5]. TERT synthesizes telomeric sequences using TERC as a template. In human normal somatic cells, TERC is constitutively expressed, whereas *TERT* expression is epigenetically silenced [6,7]. In addition, most cancer cells acquire telomerase activity by re-expressing the limiting factor TERT [7,8].

The mechanism for the regulation of *TERT* transcription has been studied for many years. In 1999, three independent groups isolated the 5’ promoter region of the *TERT* gene [9,10,11]. In the core promoter region, which exists in the proximal 260 base pair upstream from the transcription start sites and is essential for *TERT* transcription, transcription factors C-MYC and SP1 bind to the E-box (5′-CACGTG-3′) at −165 and +44 bp and five GC boxes (5’-GGGCGG-3′), respectively, to induce *TERT* mRNA expression [12]. The binding sites for the other transcription factors, such as E2F and AP-1, and an estrogen response element (ERE) for estrogen receptor α binding, have been identified in the promoter region and are involved in *TERT* transcriptional activation [12]. Another factor related to TERT regulation, CCCTC binding factor (CTCF), which functions as an insulator with cohesion by creating the higher-order chromatin loops across the genome and regulates gene expression both positively and negatively by promoting or blocking enhancer-promoter association in a position-dependent manner, respectively [13,14], has also been identified [15,16]. The phosphatidylinositol-3 kinase (PI3K)/AKT kinase pathway enhances TERT activity at the posttranslational level via TERT phosphorylation by AKT [17,18,19]. Thus, TERT expression or activity is regulated at multiple steps by various factors.

Telomeres have two major functions: Genomic sacrifice zones for the end-replication problem (i.e., prevention of loss of genomic information at chromosome ends) and chromosome end protection from DNA damage response. These functions are mainly regulated by the telomere binding protein complex, called shelterin, which is composed of six proteins: TRF1, TRF2, RAP1, TIN2, TPP1 and POT1 [20]. Telomere double-stranded DNA (dsDNA) binding protein TRF2 and single-stranded DNA binding protein POT1 are essential proteins for end protection from ATM- and ATR-dependent DNA damage responses and the following DNA repair pathways: Non-homologous end joining and homologous recombination, respectively [21,22,23,24,25]. TRF2 also protects the telomere ends by regulating the formation of a higher order telomere loop structure called t-loop [26,27,28,29]. The t-loop is formed by the invasion of a single-stranded G-overhang (G-tail, 3’-overhang) at telomere ends into double strand telomeric DNA, which prevents DNA ends from being recognized by the DNA damage response machinery and telomerase. TRF1 has DNA bending activity, which contributes to t-loop formation [30]. Other functions of TRF1 are to promote telomere replication at the S phase of the cell cycle [31] and negatively regulate telomerase through recruitment of TIN2, which tethers TPP1-POT1 heterodimer to single-stranded G-overhang [32,33,34,35]. TPP1-POT1 regulates telomerase activity both positively and negatively. POT1 limits telomerase access to G-overhangs by binding to single-stranded DNA [36], whereas TPP1 interacts with telomerase to promote telomerase processivity [4,5,37]. In addition, cell cycle-dependent phosphorylation of TPP1 is required for the TPP1-TERT interaction [38,39].

In this review, we summarize the latest knowledge obtained via whole genome analysis regarding telomere length regulation, mainly focusing on TERT point mutations and the regulatory mechanism of TERT expression. Furthermore, we summarize the rationality for the maintenance of shortened telomeres in cancer and discuss the potential utility of telomere length as a prognostic biomarker.

## 2. TERT Promoter Mutations in Cancer

Employing advanced genome sequencing technology, two different groups unraveled non-coding mutations in *TERT* promoter in melanoma. Horn’s group and Huang’s group discovered point mutation in the promoter at −124 (C > T) and −146 base pairs (C > T) from the transcription start site (TSS) (also termed C228T and C250T as these positions are at chromosome 5, 1,295,228 C > T and 1,295,250 C > T, respectively) in sporadic melanoma [40,41]. Furthermore, Horn et al. discovered a T > G point mutation in the promoter at −57 base pairs from TSS of *TERT* in familial melanoma [40]. These mutations generate novel consensus binding motifs for E-twenty-six (ETS) transcription factor (GGAA, reverse complement) in the *TERT* promoter, leading to upregulation of *TERT* mRNA expression. In ETS family proteins, ETS1 and GA-binding protein transcription factor α (GABPA) and β1 (GABPB1) dimers are specifically recruited to the de novo ETS binding motifs in the *TERT* promoter, which increases telomerase enzymatic activity and telomere elongation and is correlated with poor prognosis in urothelial cancer [42,43]. These *TERT* promoter mutations are currently the most common non-coding somatic mutations in cancer and are present in many types of cancers, including melanoma (67%), glioma (51.1%, specially 83.3% in primary glioblastoma, which is the most common and aggressive type of brain tumor), myxoid liposarcoma (79%), osteosarcoma (4.3%), hepatocellular carcinoma (44%), urothelial carcinoma (50.8%), squamous cell carcinoma (14.4%), medulloblastomas (21%), ovarian clear cell carcinoma (15.9%), thyroid cancer (10%), and bladder cancer (59%) [40,41,44,45,46].

The percentage of *TERT* promoter mutation is low in sarcomas except for myxoid liposarcoma [44,47] because 20 to 65% of sarcoma activates ALT but not telomerase to elongate telomeres [48]. In accordance with this observation, *TERT* promoter mutations are mutually exclusive with the mutations in α-thalassemia/mental retardation syndrome X-linked (ATRX), death domain associated protein (DAXX) or switch/sucrose non-fermentable (SWI/SNF) related, matrix associated, actin dependent regulator of chromatin, subfamily a, member 1 (SMARCA1), which are chromatin remodeling proteins associated with ALT pathway activation [49,50,51,52]. In addition, ARID1A and PIK3CA mutations also tend to be mutually exclusive with *TERT* promoter mutations in ovarian clear cell carcinoma or glioma [53,54]. These proteins regulate telomerase activity epigenetically or post-translationally. SWI/SNF chromatin remodeling protein ARID1A suppresses *TERT* expression by binding to TSS via the transcriptional repressor SIN3A and accumulates histone H3 lysine 9 trimethylation (H3K9me3) [55]. Therefore, ARID1A mutation causes *TERT* transcription activation. The PIK3CA/AKT/NF-kB pathway activates TERT activity at the protein level via TERT phosphorylation by AKT in breast and ovarian cancers [17,18,19].

In contrast to genes with mutual exclusivity, whole genome sequencing analysis has also revealed a simultaneous gene mutation with *TERT* promoter mutations. BRAF V600E mutations often co-occur in melanoma and thyroid cancer with *TERT* point mutations [40,41,56]. The BRAF-ERK kinase pathway activates ETS family proteins in melanoma, thyroid cancer and glioma [57,58]. In melanoma and papillary thyroid carcinoma, ERK activated by mutated BRAF induces direct phosphorylation of ETS1 [59] or phosphorylation of another transcription factor FOS followed by GABPB transcriptional activation [60]. Papillary thyroid carcinoma patients with these double mutations indicate poorer prognosis and reduced survival rates compared with those with single mutations [56]. Thus, the telomere elongation pathway functions dependently or independently of *TERT* promoter mutations. In some cases, further enhancement of telomerase activity by other gene mutations occurs in the process of tumorigenesis [59].

## 3. Epigenetic Regulation of TERT Expression

*TERT* expression is regulated not only by transcription factors but also by epigenetic status. DNA methylation in the *TERT* promoter region has been studied for two decades [61,62]. In contrast to the canonical function of DNA methylation as in gene silencing, it has been reported that methylation levels at the *TERT* promoter region are correlated with *TERT* expression levels [61,63]. DNA methylation levels in the *TERT* promoter region are altered between specific positions in the region. For example, the position between approximately −600 and −200 bp from TSS is hypermethylated, whereas the area from −200 to +150 bp is relatively methylated at lower levels [15,64,65]. Among the low methylation regions, methylation levels at −200 to −100 are reduced compared with the region from +1 to +100 position because the region from −200 to −100 includes E-box and GC box called the core promoter region to which C-MYC and SP1 bind to activate *TERT* transcription. A CCCTC binding factor (CTCF) binding motif exists in TSS (+1 to +100 region) and also in an enhancer region at approximately 4.5 kb upstream from TSS sites [15,16]. Whereas the loop structure which connects between enhancer and promoter by recruitment of CTCF in the enhancer region positively regulate *TERT* mRNA expression, the formation of loop structure at TSS suppresses downstream *TERT* transcription [13,14,16]. Given that recruitment of CTCF is disrupted by DNA methylation, DNA methylation at TSS contributes to promoting *TERT* mRNA expression [15]. Whole genome sequencing has also demonstrated that *TERT* expression is positively correlated with methylation levels at the *TERT* promoter region and is negatively correlated with gene body methylation level [66].

On the other hand, Stern et al. demonstrated that the methylation level at the −600 position is inversely correlated with *TERT* transcription. In fact, DNA methylation levels on the *TERT* promoter are different from each allele because monoallelic *TERT* promoter mutations occur in some cancer cells [67,68]. In cancer cells with monoallelic mutations, DNA methylation was present at higher levels in the wild-type allele compared with the mutated allele. Then, the histone methyltransferase for histone H3 lysine 27 trimethylation (H3K27me3), EZH2, is recruited to methylated DNA via polycomb repressive complex 2 (PRC2) to deposit the silencing histone mark exclusively on the wild-type allele. On the other hand, the active histone mark H3K4me2/3 accumulates on the mutated allele of the same promoter region, resulting in *TERT* expression exclusively from the mutated allele [65].

GABPA1 recruitment to the *TERT* promoter via point mutations also contributes to changes in epigenetic histone marks on the promoter region. Akincilar et al. reported that the introduction of *TERT* promoter mutations at −146C (−146C > T) in colorectal cancer HCT116 cells with the wild-type *TERT* promoter by CRISPR/Cas9 alters the chromatin status of the *TERT* promoter to an active conformation. In contrast, restoration of the promoter mutation to the wild-type sequence or depletion of GABPA in melanoma and glioblastoma cell lines enables silencing of the histone mark, suggesting that GABPA binding to *TERT* promoter is a key step to change the chromatin status [69]. Interestingly, GABPA recruitment to the *TERT* promoter induces long-range chromosome interaction with a region 300-kb upstream from *TERT* promoter called T-INT. Long-loop formation occurs in telomeres as well. Telomere position effect-over long distances (TPE-OLD) is another example of transcriptional regulation via the interaction of telomeres with chromosomal regions far from telomeres (see below) [70]. *TERT* is also regulated by this TPE-OLD. In this case, *TERT* expression is suppressed by loop formation with telomere through the accumulation of silencing chromatin marks [71]. The detailed mechanism is still unknown, but the histone mark on the interacted region might influence the chromatin condition on the other side (telomere is normally silent [72]).

## 4. Telomere Shortening in Cancer

Although cancers with telomerase activation acquire the ability for telomere elongation, it has been reported that the telomere length of prostate cancers is shorter compared with normal tissues [73,74]. Recently, Barthel et al. examined telomere length of 18,430 samples across 31 types of cancer cohorts using whole genome sequencing or whole exome sequencing data from The Cancer Genome Atlas (TCGA) [66]. They demonstrated that 70% of cohorts exhibit shorter telomeres compared with normal samples and the remaining 30% are regulated by ALT or suspected to be [66]. This finding is also demonstrated by fluorescence in situ hybridization staining of both nevus and melanoma sections [75].

In the well-established tumorigenesis model, telomeres in human somatic cells gradually become shortened with each cell division. After 50 to 60 cell cycles, cells with shortened telomeres provoke replicative senescence by chromosomal instability and p53 activation, which is induced by the DNA damage response according to telomere shortening [76,77,78]. However, some cells that can overcome senescence by the acquisition of genetic mutations in p53 or other checkpoint proteins continue to proliferate; thus, telomeres become critically short, and apoptosis is subsequently induced (crisis) [79,80]. At this point, a minor population of the cells that activate telomerase (or ALT pathway) acquires immortality and proceeds to carcinogenesis [79].

Chiba et al. examined the ability of *TERT* point mutants to maintain telomeres through cell division using human embryonic stem (ES) cells [75]. They introduced *TERT* promoter mutation in ES cells, differentiated these cells into fibroblasts and inactivated checkpoint proteins, such as P53 and CDKN2A. As a result, ES cells with both *TERT* point mutation and checkpoint inactivation acquired an immortalization phenotype. Of note, despite *TERT* expression, telomere length was shortened in approximately 70 cell cycles, which is near the crisis phase. Thereafter, TERT expression and telomerase activity emerged for an unknown reason, which stabilized telomere length. Indeed, *TERT* promoter mutations increase gene expression by only two- to four-fold [41,81]. In addition, quantification of *TERT* mRNA copy number revealed that there are less than 5 copies/cell in most telomerase-positive cancers [82].

Xi et al. also reported that TERT is co-localized only at 5–7% of telomeres by TERT immunofluorescence, in which an amino terminal Flag-SNAP-tag sequence is integrated by CRISPR/Cas9 genome editing [83]. This observation is consistent with previous reports that telomerase prefers to elongate the shortest telomeres [84,85,86]. These observations suggest that the extent of TERT expression and telomerase activation immediately after the acquisition of *TERT* promoter mutations are quite low to elongate all telomeres, and telomerase needs to choose the shortest telomere to elongate, resulting in gradual shortening of bulk telomere length. Then, after telomeres are shortened, TERT activity increases to sufficient levels to stabilize telomere length.

In *Saccharomyces cerevisiae*, telomerase is activated by extremely shortened telomeres depending on the activation of TEL1, which is a human ataxia telangiectasia mutated (ATM) orthologue [87]. Although it is not known whether this process occurs in human cells, ATM, ataxia telangiectasia and Rad3-related (ATR), and DNA-PK phosphorylate AKT upon induction of the DNA damage response to prompt DNA repair, apoptosis, and cell cycle arrest [88]. Given that TERT is a target of AKT, DNA damage response to telomere shortening might activate Akt, which subsequently phosphorylates TERT and increases telomerase activity.

TERT expression and telomerase activity are regulated by an indirect way as well. *TERT* gene consists of 16 exons in 42 kb of gene body at chromosome 5p15.33 and 22 splicing variants containing well-examined variants, α- and β-splicing variants, have been identified up to date [89]. None of these variants except for the full length have the enzymatic activity [90] and α-, β- and γ-splicing variants can act as dominant negative variants [91,92,93]. α-, β- and γ-splicing variants lack 36 base pairs in exon 6 (α-splicing variant), exons 7 and 8 (β-splicing variant) and exons 11–12 (γ-splicing variant), which exist in the reverse transcriptase domain, respectively. In many types of cancer cells, β-splicing variant is expressed as a major splicing variant [82,94]. In breast cancer patients with co-expression of the full length and β-splicing variants, the patients with higher β-splicing variant expression showed decreased telomerase activity [95]. Interestingly, during the development of kidney, telomerase activity disappears before the expression of the full-length mRNA is downregulated to undetectable levels and only the splicing variant is left. At this point, splicing variants become dominant against the full length, resulting in suppression of telomerase activity [96,97]. These observations suggest that the expression of alternatively spliced *TERT* mRNA regulates the expression level of the full length *TERT* and regulate telomerase activity.

We also should mention the expression levels of shelterin proteins in cancer. Previous clinical studies have reported that expression levels of shelterin genes, such as TRF1, TRF2, TIN2, and, in some cases, POT1, are elevated in many types of cancer as compared to the noncancerous tissues [98,99]. Also, it has been reported that TRF1, TRF2 and TIN2 expression levels are correlated with the progression level of cancers [100,101,102]. In addition, telomeres in cancer are shorter than those in normal tissues, which is consistent with the next generation sequencing (NGS) data, and cancers with short telomeres show poor prognosis [101,102,103]. Interestingly, high expression of these shelterin genes and TERT or even telomerase activity is inversely correlated with telomere length [101,104]. Considering the shelterin function as a negative regulator of telomerase, it is possible that the increased expression of shelterin proteins restricts telomerase activity, leading to the maintenance of short telomeres, which subsequently contribute to cancer progression.

In the process of tumor development, various mutations are introduced in the cancer cell population, causing intratumor heterogeneity. Recent single cell analyses by NGS have shown that the genetic variance is generated among the cells in a tumor [105,106,107]. Huang et al. have reported the increased telomerase activity in the heterogeneous population of T-cells at a single cell level [108]. They showed that upon mitogen stimulation, not all but only a small subpopulation of T-cells reactivate telomerase and preferentially elongate short telomeres. It is possible that there are various cell subpopulations with long to short telomeres during the course of cancer development. Because telomeres become shortened by cell proliferation, only the cells with shortest telomeres may increase telomerase activity to avoid crisis and maintain short telomeres.

## 5. Potential Advantage of Shortened Telomeres in Cancer

Given that telomerase-mediated telomere elongation is important for the infinite proliferation of TERT-positive cancer cells, genetic or pharmacological inhibition of telomerase activity in cancer cells induces gradual shortening of telomeres and eventual cell senescence or apoptosis [109,110,111]. Theoretically, the anticancer effect of telomerase inhibition would emerge earlier in cancer cells with shorter telomeres. In fact, short telomere length could be a predictive biomarker of telomerase inhibitors [112,113]. Furthermore, a clinical study has indicated that the telomerase inhibitor imetelstat increases median progression-free survival and overall survival in patients with non-small cell lung cancer with short telomeres [114]. These observations suggest that longer telomeres would be more advantageous for cancer cells if telomerase-mediated telomere elongation is abrogated. As described above, however, cancer cells often maintain their telomeres at shorter lengths compared with normal tissues.

As a well-established mechanism for limited telomere elongation, telomere-bound shelterin complexes exert a negative feedback effect on telomerase access to telomeres (so-called “protein-counting” mechanism) [115,116]. *TERT* transcripts may also inhibit telomerase function because the splicing variants work as dominant negative proteins, as described above. The second interpretation is that the length does not matter if the capping function by t-loop formation is intact. Third, moderate genomic instability elicited by shortened telomeres might be advantageous to cancer evolution [117]. In fact, induction of chromosomal instability via the telomeric DNA damage response followed by end-to-end fusions promotes oncogenic transformation [74].

We previously addressed whether it is advantageous for cancer cells to maintain short telomeres [118]. First, we identified cancer cell lines that maintained very short telomeres (prostate PC-3, stomach MKN74 and breast HBC-4 cancer cells) and elongated their telomeres by TERT overexpression. Subcutaneous injection of the resulting cells into nude mice led to the formation of xenograft tumors at comparable sizes irrespective of telomere length. Intriguingly, however, the xenografts derived from cancer cells with longer telomeres exhibited more differentiated tissue phenotypes, such as the formation of duct-like structures and the reduced expression of N-cadherin and vimentin, both of which are mesenchymal cell markers and associated with poor prognosis in cancer [119]. Furthermore, although xenograft tumors derived from cancer cells with short telomeres upregulate the expression of type I interferon (IFN) signaling-related genes (Interferon Stimulated Genes, ISGs), tumors from cells with longer telomeres do not upregulate those genes [118].

ISGs expression is regulated by signal transducer and activator of transcription 1 (STAT1), which is a transcription factor activated by IFN. This IFN/STAT1 pathway exerts an antiviral and antitumor activity by inducing cell cycle arrest or apoptosis [120,121,122], whereas it is also involved in tumor progression. The reason for these two opposite functions of IFN/STAT1 pathway remains elusive but it might be due to the difference of tumor types and genetic background, such as mutations of apoptosis- and cell cycle-related genes. Heterogeneity in the tumor might also affect the cellular response to IFN/STAT1. STAT1 is overexpressed in many types of cancers, including leukemia, breast cancer, squamous cell carcinoma of the head and neck (SCCHN), glioma, renal cell carcinoma, prostate cancer and soft tissue sarcoma [123,124,125,126]. STAT1 overexpression shows resistant to radiation and anticancer drugs and metastasis [125,127,128,129,130,131,132]. Breast cancer cells with high expression of CD44, one of cancer stem cell markers, express higher levels of STAT1 compared with cells with low CD44 expression [133]. One of STAT1-target gene, ISG15, a ubiquitin-like protein, is also involved in the regulation of the expression of stemness-related genes, cell growth, cell migration, and tumorigenicity in pancreatic ductal adenocarcinoma, hepatocellular carcinoma and breast cancers [134,135,136]. In accordance with these observations, high expression of STAT1 and its target genes (e.g., ISG15, ITI44, MX1 and OAS1) is associated with lower survival rates in high-grade glioma [132,137]. In glioblastoma, more than 70% of the cases has the *TERT* promoter mutation. One of the glioblastoma subtypes, proneural subtype, has features of high expression of platelet derived growth factor receptor alpha (PDGFRA) or isocitrate dehydrogenase 1 (IDH1) gene mutation [138]. WGS analysis showed that IDH1 mutation is mutually exclusive with *TERT* promoter mutation and partly associated with activation of the ALT pathway [138,139,140,141]. Moreover, the patients of glioblastoma with IDH1 mutation and ALT activation, which is associated with longer telomeres, showed better clinical outcome than those with ALT-negative tumor [140,142]. Thus, a majority of glioblastoma containing proneural subtype maintains shorter telomeres by *TERT* promoter mutation although there are some glioblastoma with longer telomeres by ALT activation. Given that alteration of telomere length affects the tissue morphology of the tumors and expression of cancer-associated genes, shortened telomeres might contribute to tumor malignancy through reprograming gene expression signatures.

## 6. Regulation of Gene Expression by Telomeres

Canonical telomere function involves the protection of chromosome ends and acts as sacrifice sequences for the end replication problem as mentioned above. In addition, telomere length affects the expression of genes adjacent to the telomeric region, which is called the telomere position effect (TPE). TPE was first identified in *Saccharomyces cerevisiae* by Gottschling et al., who found that expression of RNA polymerase II gene inserted immediately adjacent to telomere locus was suppressed [143]. This phenomenon is caused by silent chromatin conformation of telomeres. Representatives of the genes that are affected by TPE include ISG15 located at human chromosome 1p36.33, which is 1 Mb from telomere and exhibits higher expression in older-aged cells [144]. Robin et al. reported that TPE affects not only genes near telomeres but also those far from telomeres up to 10 Mb by means of long-range loop formation [70]. This atypical TPE is called telomere position effect over long distances (TPE-OLD). Hi-C (chromosome capture followed by high-throughput sequencing) analysis has revealed that some genes, such as *DSP (desmoplakin)*, which is located 7.5 Mb away from telomeres, interact with the subtelomeric region by chromosome loop formation in a telomere length-dependent manner [70]. As another example of TPE-OLD, the shelterin component TRF2 tethers the telomere and interstitial telomere repeats to generate a long-range chromatin loop. These interstitial telomere repeats exist 100-kb downstream of the *TERT* gene and form a TRF2-dependent chromosome loop with the telomere to suppress TERT gene expression [71]. Given that 2920 interstitial telomere repeat sequences were identified in the human genome by whole genome sequencing [71], TPE-OLD might affect genome-wide gene expression more broadly than expected. Furthermore, Mukherjee et al. have recently reported telomere length-dependent, genome wide transcriptional regulation by TRF2 [145]. TRF2 is enriched at promoter regions of several genes far from telomeres in the cells with shorter telomeres and dissociates from there by telomere elongation. RE-1 silencing transcription factor (REST) and lysine-specific demethylase 1 (LDS1) are recruited to the TRF2-bound promoter regions, resulting in suppression of gene expression by adding the silencing histone marks [145].

Telomere length-dependent regulation of gene expression may also involve telomeric non-coding RNA called TERRA, which is associated with telomere end protection, maintenance of chromatin structure and telomere length regulation [146,147,148,149,150]. Telomere elongation by TERT overexpression leads to enhanced TERRA signals, due to the increased number of telomeric tracts in a single TERRA molecule. Thus, enhanced TERRA signals are associated with decreased expression of ISGs in telomere-elongated cancer cells [151]. TERRA-mimicking oligonucleotides repress ISG expression in cancer cells with short telomeres. These observations suggest that cancer cells with longer telomeres upregulate TERRA signals, which subsequently downregulate ISG expression. On the other hand, recent reports have shown that extracellular TERRA included in the exosome vesicles is amplified by telomere stress, and induces inflammatory signaling in recipient cells [152,153]. Differential behavior of TERRA might be due to where TERRA exists. Secreted TERRA in exosomes may activate inflammation by the activation of Toll-like receptors in the recipient cells whereas intracellular TERRA suppresses an innate immune response, which is activated in the process of tumor formation. Although the functional mediator that binds TERRA and represses ISG expression remains unknown, this phenomenon depends on G-quadruplex formation in TERRA and in TERRA-mimicking oligonucleotides [151]. It is possible that cancer cells may prefer shortened telomeres, wherein TERRA downregulation allows ISG expression for cancer progression.

## 7. Conclusions and Future Perspectives

**Figure 1:**
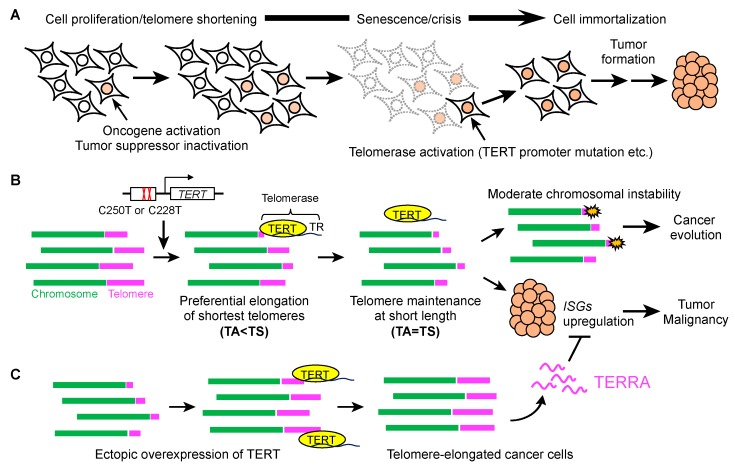
Bimodal implication of telomere shortening in cancer development; (**A**) human somatic cells shorten their telomeres at each cell cycle. Activation of oncogenes and inactivation of tumor suppressor genes lead to dysregulated cell proliferation, which further enhances telomere shortening. A critically shortened telomere causes senescence or crisis of a cell, whereas telomere reverse transcriptase (TERT) expression mainly via promoter mutation (C228T or C250T) results in telomerase activation and tumor formation; (**B)** in the initial step of carcinogenesis, the level of telomerase activity (TA) is insufficient to prevent telomere shortening (TS) and indicates as “TA < TS”. Under these conditions, telomerase preferentially elongates the shortest telomeres, but the level of the enzyme activity is insufficient to maintain the bulk telomere length. After several cell cycles, additional factors upregulate telomerase activity, and the bulk telomere length is maintained short in an equilibration between TA and TS (“TA = TS”). In a tumor mass, cancer cells with short telomeres upregulate interferon-stimulated genes (ISGs), which presumably contribute to the tumor malignancy. Furthermore, shortened telomeres facilitate cancer evolution by causing moderate chromosomal instability; (**C**) in experimental settings, ectopic overexpression of TERT induces telomere elongation in cancer cells, which is followed by TERRA upregulation and repression of ISG expression. Because TERRA-like oligonucleotides repress ISG induction, shortened telomeres, which provide reduced TERRA levels, may be more advantageous for cancer.

Identification of *TERT* promoter mutations has triggered a remarkable improvement of our understanding of the mechanism for *TERT* transcriptional activation and the relationship between *TERT* promoter mutations and other gene mutation to regulate TERT expression and telomere length in a cooperative or mutually exclusive manner. These observations have established the importance of telomere maintenance for carcinogenesis and its malignant progression. Nevertheless, most cancers exhibit shorter telomeres compared with normal tissues despite the risk of crisis, apoptosis or senescence, due to excessive telomere shortening. One rational answer might be that maintaining shortened telomeres causes activation of specific genes, such as ISGs, which are required for cancer progression (Figure 1).

Accumulating evidence has demonstrated that short telomeres correlate with increased cancer malignancy, and telomere length in cancer would be useful as a prognostic biomarker or a risk predictor. Some reports have shown the association of telomere length with a poor prognosis of cancer by monitoring telomere length in blood cell DNA as a surrogate biomarker [154,155,156]. Furthermore, exosome-derived DNA or circulating cell-free DNA (cfDNA) might be more useful to examine the utility of telomere length as a biomarker because cancer-derived DNA exists in those samples. For example, Wan et al. reported the association of telomere length measured by using circulating serum DNA with the risk of hepatocellular carcinoma in hepatitis B virus patients [157]. Meanwhile, it has been also reported that length of the telomeric G-tail (3′-overhang) instead of telomere length, is a better biomarker for predictions of disease risk [158,159]. TERRA also might become a useful biomarker because it is included in exosomes as above mentioned. Further investigations will reveal the relationship between telomere length and gene expression regulated by telomeres and tumor progression in more detail, and a deeper understanding of telomere length as a biomarker will ensure its utility in cancer precision medicine and prevention.
